# Beyond the bladder: incontinence-specific, physical, and psychological contributors to sexual function in women with urinary incontinence

**DOI:** 10.1093/jsxmed/qdag175

**Published:** 2026-06-25

**Authors:** Nancy Yang, Abigail Shatkin-Margolis, Leslee L Subak, Margaret Chesney, Nadra E Lisha, Michael Schembri, A Lenore Ackerman, Alison J Huang

**Affiliations:** School of Medicine, University of California, San Francisco, San Francisco, CA 94143, United States; Department of Obstetrics, Gynecology, and Reproductive Sciences, University of California, San Francisco, San Francisco, CA 94158, United States; Department of Obstetrics and Gynecology, Stanford University, Palo Alto, CA 94304, United States; Division of Prevention Science, Department of Medicine, University of California, San Francisco, San Francisco, CA 94143, United States; Division of Prevention Science, Department of Medicine, University of California, San Francisco, San Francisco, CA 94143, United States; Division of General Internal Medicine, Department of Medicine, University of California, San Francisco, San Francisco, CA 94143, United States; Departments of Urology and Obstetrics and Gynecology, University of California, Los Angeles, Los Angeles, CA 90095, United States; Division of General Internal Medicine, Department of Medicine, University of California, San Francisco, San Francisco, CA 94143, United States

**Keywords:** sexual behavior, urinary incontinence, depression

## Abstract

**Objective:**

Urinary incontinence (UI) can interfere with sexual activity in women, yet sexual function may be shaped by a more complex interplay of physical and psychological factors in women with frequent UI. We aimed to assess the associations of incontinence-specific, physical function-related, and psychological factors with multidimensional sexual function in midlife and older women with UI.

**Methods:**

We conducted ancillary analyses of sexual function data from a multicenter randomized trial of a pelvic floor yoga intervention versus physical conditioning intervention in women aged 45 years or older with at least daily UI. Urinary incontinence symptoms were assessed using validated voiding diaries at baseline and 6, 12, 24, and 36 weeks. Physical and psychological function were assessed by standardized performance tests and questionnaires (including the Patient-Reported Outcomes Measurement Information System physical function profile short-form, 2-minute step testing, Center for Epidemiological Studies Depression scale, and Hospital Anxiety and Depression Scale-Anxiety Subscale). Sexual function was evaluated using the Pelvic Organ Prolapse/Incontinence Sexual Questionnaire, IUGA-Revised scales for condition-specific impact on sexual activity (CS), condition-specific impact on sexual quality (CI), and global sexual quality rating (GQ). Repeated-measures mixed linear regression models were used to compare mean sexual function scores across different incontinence-specific, physical, and psychological factors, while adjusting for the influence of all other factors.

**Results:**

Of the 240 participants, 129 (54.7%) were sexually active at baseline. Among sexually active participants, UI frequency was associated with worse mean CS scores (90.9 vs. 86.9 vs. 86.2, *P* = .03), depression symptoms were associated with worse CS scores (89.8 vs. 86.2, *P* = .049), and anxiety symptoms were associated with worse CI scores (82.0 vs. 77.1, *P* = .04). Among non-sexually active participants, depression symptoms were associated with worse GQ (35.0 vs. 44.8, *P* = .01) and CI scores (7.9 vs. 18.5, *P* = .01), and lower physical function was associated with worse CI scores (18.1 vs. 12.2 vs. 9.3, *P* = .04). No significant differences in sexual function were associated with clinical type of UI (eg, urge-dominant, stress-dominant) after adjustment for physical or psychological factors.

**Conclusion:**

Even among women with daily UI, incontinence-specific factors were not major determinants of sexual function, nor was their physical function status. Instead, variations in sexual function were more consistently associated with psychological function. Recognizing sexual function and UI concerns as distinct processes and addressing overlapping psychological symptoms may offer the greatest value in women with poor sexual function and UI.

Clinical trial registration number**:** NCT03672461.

## Introduction

Urinary incontinence (UI) affects up to half of midlife and older women^1^ and is associated with self-reported difficulty engaging in sexual activity or having satisfying sexual relationships.^2^ Women with UI are more likely to be sexually abstinent and experience reduced sexual desire, arousal, and satisfaction compared to continent women.^3-5^ Independent from UI, poor sexual function has also been shown to predict depressive symptoms and worsened perceived quality of life.^6^^,^^7^

Nevertheless, while UI can contribute to women’s sexual problems, sexual dysfunction in midlife and older age is more likely to be multifactorial, influenced by a range of physical, psychological, and relational factors. For example, depression and anxiety have been reported to be independently associated with worse sexual function in older women.^6^^,^^8-10^ Overall health status and physical function may also contribute to sexual dysfunction, as they can influence motivation and capacity to engage in sexual activity.^9^^,^^11^ However, the relative importance of these factors in predicting sexual function outcomes among women with UI remains unclear. Furthermore, many prior studies linking UI and sexual dysfunction have been cross-sectional and have not incorporated repeated assessments of symptoms over time.

To address these gaps, we present prospective research examining incontinence-specific features, physical function, and psychological factors associated with sexual function in a diverse sample of midlife and older women engaging in complementary behavioral interventions for UI. We evaluated the strength of associations between these factors and multiple dimensions of sexual function among both sexually active and non-sexually active women with UI. Our goals were to provide deeper insight into the potentially independent implications of UI, physical function, and psychological function for sexual function in women with UI and identify the factors with the strongest implications for whether women have satisfying sexual experiences.

## Materials and methods

### Setting/participants

We present secondary, post-hoc observational analyses of prospective data from the Lessening Incontinence with Low-impact Activity (LILA) trial, a randomized, multicenter, parallel-group superiority trial comparing a therapeutic pelvic yoga program versus non-specific physical conditioning program for UI in ambulatory midlife and older women. Participants were recruited from northern California, USA, by research staff based at 3 study sites from 2019 to 2022.^12^ To be eligible for the study, participants had to be at least 45 years of age, report at least daily stress-type, urgency-type, or mixed stress- and urgency-type incontinence for at least 3 months. Candidates were excluded if they had major mobility limitations, pregnancy in the past 6 months, current urinary tract infection or hematuria, history of recurrent urinary tract infections, major neurologic or pelvic conditions, prior anti-incontinence or urethral surgery, or other pelvic surgery in the past 3 months. Participants were required to be English-speaking, as all questionnaires were administered using validated English-language instruments.

Participants were randomized in a 1:1 ratio to a therapeutic pelvic floor yoga program or non-specific physical conditioning program using a computer-generated allocation sequence with concealed assignment.^12^ Randomization was stratified by clinical incontinence type and study site. Each intervention involved twice weekly group classes for 12 weeks. After the end of the intervention program, participants were encouraged to continue practicing yoga or physical conditioning exercises at home.

The sample size of 240 participants was chosen to detect a 20% difference in reduction in UI frequency between intervention groups. All participants provided written informed consent prior to screening. All study procedures were approved by our institutional review board. This secondary analysis was reported in accordance with the STROBE guidelines.

### Sexual function measures

Sexual function was examined before intervention initiation at baseline, and again at 6, 12, 24, and 36 weeks using the Pelvic Organ Prolapse/Incontinence Sexual Questionnaire, IUGA-Revised (PISQ-IR), a structured, validated, 20-item self-report measure of sexual function designed for women with pelvic floor disorders. The PISQ-IR has previously been shown to be a reliable and responsive measure of sexual function in clinical studies in both sexually active and non-sexually active women with UI.^13^ For this analysis, we selected a subset of PISQ-IR subscales that were most relevant to overall sexual function and the impact of pelvic floor symptoms.^14^ For sexually active women, we focused on the following PISQ-IR subscales: SA-CS (assessment of condition-specific impacts on activity), SA-GQ (global quality rating of sexual quality), SA-CI (condition-specific impact on sexual quality). On these subscales, higher scores indicate better sexual function. For non-sexually active women, we focused on the following PISQ-IR subscales: NSA-CS (condition-specific reasons for not being active), NSA-GQ (global quality rating of sexual quality), and NSA-CI (condition impact on sexual quality). On these subscales, higher scores reflect greater negative impact of pelvic floor conditions on sexual inactivity.

### Other measurements

Incontinence-specific variables were assessed using a combination of interviewer-administered questionnaires and validated participant-administered voiding diaries. Validated voiding diaries were kept by participants over a 3-day period at baseline and 6, 12, 24, and 36 weeks, in which they documented and characterized all incontinence episodes. Trained analysts abstracted diary data to describe frequency of incontinence (average number of episodes per day) and predominant clinical incontinence type (stress-predominant, urgency-predominant, or mixed).^15^^,^^16^

Perceived physical function was assessed at baseline, 6 weeks, and 12 weeks using the Patient-Reported Outcomes Measurement Information System (PROMIS) Adult Physical Function Profile short-form, a self-administered questionnaire assessing functioning of the upper extremities, lower extremities, and central regions (neck, back).^17^ Objective assessment of physical function included the 2-minute step test, a standardized test of aerobic endurance in which participants marched in place as many times as possible over 2 min, with scores reflecting the total number of times participants were able to raise their left knee to their iliac crest within 2 min.^18^

Psychological variables were assessed using multiple validated, self-administered questionnaires at baseline and 6, 12, 24, and 36 weeks. Depression symptoms were assessed using the 20-item version of the Center for Epidemiologic Studies Depression Scale (CES-D).^19^ Scores range from 0 to 60 and higher scores indicate greater depression symptom burden. The Cognitive anxiety was assessed using the anxiety subscale of the Hospital Anxiety and Depression Scale (HADS), scaled from 0 to 21, with higher scores indicating greater anxiety.^20^^,^^21^ The HADS anxiety subscale was selected to measure cognitive anxiety because it assesses fear, tension, and worry while minimizing overlap with physical and somatic symptoms.

Demographics variables and medical and urogynecologic history were assessed via structured interviewer-administered screening questionnaires. Sexual orientation was determined using a multiple-choice question ahead of the PISQ-IR questionnaire. All prescription and over-the-counter medications were reviewed and recorded at baseline. Sexual activity was assessed using the first question of the PISQ-IR, which asks whether participants are “not sexually active at all” or “sexually active with or without a partner.”

### Statistical analysis

Descriptive analyses examined the distribution of participant characteristics, including PISQ-IR domain scores, at baseline. Next, we developed repeated-measures multivariable linear regression models to evaluate the associations of UI-specific, physical function, and psychological factors with PISQ-IR domain scores. To maximize interpretability of findings for patient and clinician readers, we examined participants within clinically interpretable categories of UI frequency, psychological function, and physical function. In particular, mean incontinence frequency was categorized as fewer than 1 episode per day, at least 1 but fewer than 3 episodes per day, and at least 3 episodes per day, following the precedence of previous studies.^22^^,^^23^ For psychological measures, we used thresholds of 16 or greater for the CES-D and 11 or greater for the HADS anxiety subscale, as these are previously-established thresholds corresponding to higher risk of clinical depression^19^ and moderate to severe anxiety symptoms,^24^ respectively. For PROMIS Adult Physical Function Profile and 2-minute step test scores, no clear clinically-based categories have been established. Therefore we compared participants across tertiles, following previous works,^25^^,^^26^ to facilitate interpretation of adjusted mean PISQ-IR scores across lower-, middle-, and higher-function groups.

We first examined independent associations between sexual function and each predictor variable using minimally-adjusted models that included only age and intervention assignment as covariates. Age is a well-recognized factor associated with sexual function and several of the other domains analyzed, including physical function and incontinence symptom burden. Intervention assignment was included because participants were followed within a randomized trial in which pelvic floor yoga versus physical conditioning could influence urinary symptoms, physical function, psychological function, and sexual function over time. Then, we fit a fully-adjusted model that was further adjusted with selected characteristics deemed likely to be relevant to sexual, urinary, psychological, and physical function (self-reported race, BMI, and menopausal status), in addition to all other potentially influential factors (UI type, UI frequency, PROMIS Adult Physical Function Profile score, 2-minute step test, CES-D score, and HADS anxiety subscale score). Effect estimates were calculated using least square means to report adjusted mean sexual function scores. We used all available survey responses at each timepoint; participants who were lost to follow-up or missed one or more outcome measurement timepoints contributed data from the timepoints they completed.

All analyses were performed in SAS v9.4. Statistical tests were two-sided and conducted using a significance level of 0.05. Because these were exploratory analyses, no formal adjustment for multiple comparisons was applied, to avoid obscuring potentially meaningful associations.

## Results

Of the 482 candidates screened in 2019-2022, 329 were determined to be eligible, and 240 were enrolled in the parent LILA trial, and all provided sexual function data at one or more timepoints and were included in this report. All participants identified as women, the mean age was 62.1 years (SD 8.7), and the majority self-identified as White (66.7%) and college-educated (82.9%) (Table 1). Almost two-thirds were married or partnered, and 129 (53.8%) reported being sexually active at baseline (Table 1). The number of participants contributing data at each follow-up timepoint is provided in Supplemental Table S1.

**Table 1:** Baseline participant demographic and clinical characteristics.

	**Overall (*N* = 240)**
**Demographics**	
**Age**	62.1 (±8.7)
**Race**	
Asian	40 (16.7)
Black or African American	19 (7.9)
More than one race	20 (8.3)
Other^a^	1 (0.4)
White	160 (66.7)
**Hispanic or Latina ethnicity**	32 (13.3)
**Educational attainment**	
College degree or more	199 (82.9)
Some college/AA degree	31 (12.9)
High school or less	10 (4.2)
**Relationship status**	
Married	148 (61.7)
Other/partnered	13 (5.4)
Single/divorced/widowed	78 (32.5)
**Sexual activity status**^**b**^	
Sexually active	129 (53.8)
**Sexual orientation**	
Straight (Heterosexual)	219 (91.3)
Lesbian	2 (0.8)
Queer	1 (0.4)
Questioning	3 (1.3)
Other	2 (0.8)
Decline to state	10 (4.2)
**Clinical characteristics**	
**Body mass index**	27.2 (±5.4)
**Nulliparous**	63 (26.3)
**Postmenopausal**	201 (83.8)
**History of oophorectomy**	21 (8.8)
**History of hysterectomy**	39 (16.3)
**Selected medications**	
Estrogens	31 (15.5)
Androgens	3 (1.5)
Antidepressant	32 (16)
**Incontinence characteristics**	
**Incontinence type**	
Predominantly urge (>75% urge)	91 (37.9)
Predominantly stress (>75% stress)	53 (22.1)
Mixed	95 (39.6)
**Incontinence frequency (episodes per day)**	
≥1 and <3	116 (48.3)
≥3	123 (51.3)
**Duration of incontinence (years)**	
<5	94 (39.2)
5-9	60 (23.8)
10 or more	85 (35.4)
**Physical function scores**	
**PROMIS Physical Function Profile Short-Form**^**c**^	51.2 (±6.5)
**2-minute step test**^**d**^	87.1 (±24.3)
**Psychological function**	
**CES-D score**	
≥16^e^	55 (22.9)
**HADS anxiety subscale score**	
≥11^f^	76 (31.8)

Data are presented as mean (±SD) for numeric variables or number (percentage) for categorical or binary variables.

a Participants were given the option of choosing “other.”

b Sexual activity was using the first question of the PISQ-IR. Participants choosing “sexually active with or without a partner” were considered sexually active, and participants choosing “not sexually active at all” were considered not sexually active.

c PROMIS physical function scores represent t-scores with 50 being the average for the United States general population and a standard deviation of 10.

d Scores for the 2-minute step test represents the number of times the right knee reaches the designated height during a 2-minute period. Higher scores reflect better aerobic endurance. Scores less than 65 are associated with lower levels of functional ability.

e CES-D scores greater than or equal to 16 indicate high risk for clinical depression.

f HADS anxiety subscale scores greater than or equal to 11 indicate moderate to severe anxiety symptoms.

Abbreviations: PISQ-IR, Prolapse/Incontinence Sexual Questionnaire, IUGA-Revised; PROMIS, Patient-Reported Outcomes Measurement Information System; CES-D, Center for Epidemiological Studies-Depression; HADS, Hospital Anxiety and Depression Scale.

Ninety-one (37.9%) participants reported predominantly urge UI, 53 (22.1%) reported predominantly stress UI, and 95 (39.6%) reported mixed UI (Table 1). Approximately half (51.3%) experienced at least 3 incontinence episodes per day. Mean PROMIS Physical Function score at baseline was 51.2 (SD 6.5), and mean 2-minute step test score was 87.1 (SD 24.3). 22.9% had CES-D scores ≥16, and 31.8% had HADS anxiety subscale scores ≥11. Mean baseline PISQ-IR scores are presented in Supplemental Table S2.

PISQ-IR sexual function scores did not differ significantly among participants based on clinical incontinence type in minimally-adjusted and fully-adjusted models accounting for self-reported race, BMI, menopausal status, and all incontinence-specific, physical function, and psychological factors simultaneously (Table 2). For instance, mean adjusted global quality scores among sexually active participants were 65.8 among those with stress predominant UI, 64.8 among those with urge predominant UI, and 63.2 among those with mixed UI (*P* = .52). However, greater frequency of UI was associated with worse perceived impact of UI on sexual quality among sexually active women, even after full adjustment (Table 2). Mean SA-CS scores were 86.2 for those with at least 3 episodes per day, 86.9 for those with at least 1 but fewer than 3 episodes per day, and 90.9 for those with fewer than 1 episode per day (*P* = .03).

**Table 2:** Mean Pelvic Organ PISQ-IR scores, by urinary incontinence-specific factors, in minimally-adjusted and fully-adjusted models

	**Incontinence type** ^**a**^				**Incontinence frequency (mean episodes/day)**			
**PISQ-IR domain**	Adjusted mean (95% CI)^b^			*P*	Adjusted mean (95% CI)^b^			*P*
	Stress predominant	Urge predominant	Mixed		<1	≥1 and <3	≥3	
**Minimally-adjusted model** ^**c**^								
Non-sexually active scales (*N* = 128)^d^								
NSA-CS	21.3 (16.1, 26.6)	19.8 (15.5, 24.0)	18.6 (14.1, 23.2)	.63	19.3 (14.7, 23.9)	18.5 (14.6, 22.4)	20.9 (16.2, 25.7)	.56
NSA-CI	12.9 (7.4, 18.3)	16.8 (12.3, 21.3)	17.9 (13.2, 22.7)	.22	9.6 (5.0, 14.3)	16.6 (12.6, 20.5)	20.0 (15.2, 24.8)	**<.01**
NSA-GQ	48.4 (41.9, 55.0)	53.0 (47.3, 58.6)	49.4 (43.6, 55.2)	.28	50.9 (45.0, 56.9)	50.9 (45.5, 56.2)	48.8 (42.7, 54.9)	.71
Sexually active scales (*N* = 154)^e^								
SA-CS	88.7 (86.4, 91.0)	88.9 (86.6, 91.2)	88.2 (86.1, 90.4)	.82	92.0 (90.0, 94.0)	88.2 (86.3, 90.0)	86.5 (84.2, 88.9)	**<.001**
SA-CI	84.9 (81.0, 88.8)	80.4 (76.6, 84.3)	81.2 (77.6, 84.9)	.08	85.9 (82.3, 89.4)	81.3 (78.0, 84.6)	79.6 (75.6, 83.6)	**<.01**
SA-GQ	63.6 (58.7, 68.5)	63.0 (58.1, 67.9)	62.3 (57.6, 67.0)	.70	63.4 (58.7, 68.1)	61.8 (57.3, 66.3)	64.1 (59.1, 69.1)	.24
**Fully-adjusted model** ^**f**^								
Non-sexually active scales (*N* = 125)^d^								
NSA-CS	19.4 (10.2, 28.7)	15.1 (6.4, 23.7)	14.8 (6.3, 23.3)	.40	17.1 (7.7, 26.5)	15.5 (7.2, 23.8)	16.7 (7.7, 25.8)	.84
NSA-CI	10.4 (1.0, 19.8)	15.4 (6.6, 24.3)	13.8 (5.3, 22.3)	.43	9.0 (-0.4, 18.4)	14.8 (6.5, 23.2)	15.8 (6.5, 25.1)	.24
NSA-GQ	37.6 (26.3, 48.9)	43.9 (33.0, 54.8)	38.2 (27.6, 48.8)	.12	43.6 (32.4, 54.8)	41.4 (30.9, 51.8)	34.8 (23.5, 46.0)	.06
Sexually active scales (*N* = 151)^e^								
SA-CS	87.2 (82.3, 92.2)	88.7 (83.5, 93.9)	88.1 (83.0, 93.1)	.70	90.9 (85.5, 96.4)	86.9 (82.0, 91.8)	86.2 (81.2, 91.2)	**.03**
SA-CI	82.9 (73.1, 92.8)	77.9 (67.6, 88.2)	77.8 (67.8, 87.7)	.08	81.9 (71.5, 92.4)	80.1 (70.4, 89.8)	76.6 (66.7, 86.4)	.18
SA-GQ	65.8 (53.2, 78.4)	64.8 (51.8, 77.8)	63.2 (50.5, 75.9)	.52	65.7 (52.6, 78.8)	63.3 (50.7, 75.8)	64.8 (52.2, 77.4)	.48

a Stress predominant UI was defined by reporting that at least 75% of UI episodes were stress-type. Urge predominant UI was defined by reporting that at least 75% of UI episodes were urge-type. Mixed UI was defined by reporting that neither stress-type or urge-type episodes comprised of over 75% of episodes.

b Mixed linear regression model taking into account within person correlations with repeated measures across different time points, adjusted for baseline age and intervention assignment.

c Minimally-adjusted models were adjusted for age and intervention assignment.

d For non-sexually active scales, higher scores indicate more interference or problems with sexual function. Because participants may have varied in their sexual activity status across visits, the *N* refers to the number of participants providing data on at least one timepoint.

e For sexually active scales, higher scores indicate better sexual function. Because participants may have varied in their sexual activity status across visits, the *N* refers to the number of participants providing data on at least one timepoint.

f Fully-adjusted models were adjusted for age, intervention assignment, self-reported race, BMI, menopausal status, and all other potentially influential factors (PROMIS Adult Physical Function Profile score, 2-minute step test, Center for Epidemiologic Studies-Depression scale, and HADS Anxiety Subscale).

Abbreviations: HADS, Hospital Anxiety and Depression Scale; PISQ-IR, Prolapse/Incontinence Sexual Questionnaire, IUGA-Revised; PROMIS, Patient-Reported Outcomes Measurement Information System; NSA-CS, condition-specific reasons for not being active; NSA-CI, condition impact on sexual quality; NSA-GQ, global quality rating of sexual quality; SA-CI, condition-specific impact on sexual quality; SA-CS, assessment of condition-specific impacts on activity; SA-GQ, global quality rating of sexual quality; UI, urinary incontinence.

Worse physical function was associated with worsened impact of UI on sexual quality among sexually active participants in the minimally-adjusted model (Table 3). Mean SA-CI scores were 79.3, 82.9, and 84.6 among participants in the lowest (<47.5), middle (47.5-52.5), and highest (≥52.5) tertile of PROMIS Physical Function scores, respectively (*P* = .047). However, this association was attenuated in the fully-adjusted model: mean SA-CI scores were 77.3, 79.2, and 82.1, respectively (*P* = .24). PROMIS Physical Function scores were not associated with any PISQ-IR sexual function scales in non-sexually active participants. Lower 2-minute step test performance was associated with worse NSA-CI scores in the fully-adjusted model, with means of 18.1 in the lowest tertile (<77), 12.2 in the middle tertile (77-98), and 9.3 in the highest tertile (≥98) (*P* = .04, Table 3). It was not significantly associated with any PISQ-IR scales among sexually active participants.

**Table 3:** Mean Pelvic Organ PISQ-IR scores, by physical function status, in minimally-adjusted and fully-adjusted models

	**PROMIS Physical Function Profile Short-Form**				**2 minute step test**			
**PISQ-IR domain**	Adjusted mean (95% CI)^a^			*P*	Adjusted mean (95% CI)^a^			*P*
	Lowest tertile (<47.5)	Middle tertile (47.5-52.5)	Highest tertile (≥52.5)		Lowest tertile (<77)	Middle tertile (77-98)	Highest tertile (**≥**98)	
**Minimally-adjusted model** ^**b**^								
Non-sexually active scales (*N* = 128)^c^								
NSA-CS	21.5 (17.2, 25.9)	18.7 (14.6, 22.8)	16.9 (12.0, 21.8)	.27	21.2 (16.3, 26.0)	20.2 (15.3, 25.0)	20.1 (14.8, 25.3)	.92
NSA-CI	17.7 (13.2, 22.1)	13.1 (8.9, 17.3)	15.6 (10.6, 20.7)	.15	18.9 (13.9, 23.8)	13.8 (8.8, 18.7)	13.1 (7.8, 18.4)	.14
NSA-GQ	52.0 (46.2, 57.8)	49.2 (43.7, 54.7)	49.2 (42.8, 55.5)	.52	51.4 (45.2, 57.6)	47.8 (41.8, 53.8)	49.1 (42.6, 55.5)	.39
Sexually active scales (*N* = 154)^d^								
SA-CS	88.1 (85.9, 90.2)	89.4 (87.3, 91.5)	89.9 (87.8, 92.1)	.33	89.4 (87.0, 91.9)	88.2 (86.0, 90.4)	89.1 (86.7, 91.5)	.61
SA-CI	79.3 (75.5, 83.2)	82.9 (79.2, 86.6)	84.6 (80.8, 88.4)	**.05**	81.4 (77.1, 85.8)	81.2 (77.2, 85.2)	81.5 (77.3, 85.8)	.98
SA-GQ	62.0 (57.1, 66.9)	63.4 (58.6, 68.1)	63.2 (58.3, 68.1)	.74	61.7 (56.4, 67.0)	62.8 (57.9, 67.8)	63.0 (57.6, 68.2)	.85
**Fully-adjusted model** ^**e**^								
Non-sexually active scales (*N* = 123)^c^								
NSA-CS	19.9 (11.6, 28.2)	16.7 (8.0, 25.5)	12.7 (3.2, 22.2)	.17	17.8 (9.3, 26.4)	14.9 (6.2, 23.5)	16.6 (7.5, 25.7)	.60
NSA-CI	16.3 (8.0, 24.6)	9.2 (0.3, 18.0)	14.2 (4.5, 23.8)	.07	18.1 (9.6, 26.7)	12.2 (3.3, 21.0)	9.3 (0.2, 18.4)	**.04**
NSA-GQ	41.2 (30.7, 51.6)	41.2 (30.4, 52.0)	37.4 (25.7, 49.0)	.53	42.9 (32.2, 53.6)	37.1 (26.4, 47.9)	39.7 (28.5, 50.8)	.12
Sexually active scales (*N* = 149)^d^								
SA-CS	87.5 (82.4, 92.6)	88.8 (83.6, 93.9)	87.7 (82.7, 92.7)	.67	88.0 (83.0, 93.1)	87.3 (82.2, 92.3)	88.7 (83.6, 93.8)	.59
SA-CI	77.3 (67.2, 87.4)	79.2 (69.1, 89.3)	82.1 (72.1, 92.0)	.24	79.9 (69.9, 89.9)	78.4 (68.5, 88.3)	80.3 (70.3, 90.3)	.63
SA-GQ	64.3 (51.4, 77.2)	63.8 (51.0, 76.6)	65.7 (53.0, 78.4)	.78	64.1 (51.4, 76.9)	64.8 (52.2, 77.5)	64.9 (52.0, 77.6)	.95

a Mixed linear regression model taking into account within person correlations with repeated measures across different time points, adjusted for baseline age and intervention assignment.

b Minimally-adjusted models were adjusted for age and intervention assignment.

c For non-sexually active scales, higher scores indicate more interference or problems with sexual function. Because participants may have varied in their sexual activity status across visits, the *N* refers to the number of participants providing data on at least one timepoint.

d For sexually active scales, higher scores indicate better sexual function. Because participants may have varied in their sexual activity status across visits, the *N* refers to the number of participants providing data on at least one timepoint.

e Fully-adjusted models were adjusted for age, intervention assignment, self-reported race, BMI, menopausal status, and all other potentially influential factors (urinary incontinence type, urinary incontinence frequency, Center for Epidemiologic Studies-Depression scale, and HADS Anxiety Subscale).

Abbreviations: HADS, Hospital Anxiety and Depression Scale; PISQ-IR, Prolapse/Incontinence Sexual Questionnaire, IUGA-Revised; PROMIS, Patient-Reported Outcomes Measurement Information System; NSA-CS, condition-specific reasons for not being active; NSA-CI, condition impact on sexual quality; NSA-GQ, global quality rating of sexual quality; SA-CI, condition-specific impact on sexual quality; SA-CS, assessment of condition-specific impacts on activity; SA-GQ, global quality rating of sexual quality.

In minimally-adjusted models, both depression and anxiety symptom burden were strongly associated with worse sexual function across nearly all domains (Table 4). Among sexually active women, those with CES-D depression screening scores ≥16 had worse mean SA-CS (86.5 vs. 89.9, *P* < .01), SA-CI (77.6 vs. 83.7, *P* < .01), and SA-GQ (56.6 vs. 64.6, *P* < .001) scores compared to those with CES-D scores <16. Among non-sexually active women, those with CES-D depression screening scores ≥16 had worse mean NSA-CI (23.6 vs. 13.2, *P* < .001) and NSA-GQ (54.7 vs. 48.9, *P* = .04) scores. Similarly, women with greater risk of anxiety symptoms (HADS ≥11) had worsened sexual function scores across all domains. In the fully-adjusted model, depression symptoms continued to be associated with worse sexual function in several PISQ-IR domains (Table 4). For example, participants with high depression symptom burden (CES-D ≥ 16) had an average NSA-GQ score of 44.8, compared to 35.0 among participants with low depression symptom burden (CES-D < 16) (*P* = .01), and SA-CS of 86.2 compared to 89.8 (*P* = .049). Participants with greater risk of anxiety symptoms (HADS ≥11) had a lower mean SA-CI score of 77.1, compared to 82.0 among participants with little anxiety (HADS <11) (*P* = .04).

**Table 4:** Mean Pelvic Organ PISQ-IR scores, by depression and anxiety status, in minimally-adjusted and fully-adjusted model

	**Center for Epidemiologic Studies-Depression Scale**			**HADS, Anxiety Subscale**		
**PISQ-IR domain**	Adjusted mean (95% CI)^a^		*P*	Adjusted mean (95% CI)^a^		*P*
	<16	≥16		<11	≥11	
**Minimally-adjusted model** ^**b**^						
Non-sexually active scales (*N* = 131)^c^						
NSA-CS	18.7 (15.2, 22.2)	21.4 (16.2, 26.6)	.30	17.9 (14.3, 21.4)	22.4 (18.0, 26.9)	**.04**
NSA-CI	13.2 (9.6, 16.7)	23.6 (18.3, 38.8)	**<.001**	13.3 (9.7, 16.9)	20.3 (15.7, 24.8)	**<.01**
NSA-GQ	48.9 (44.0, 53.8)	54.7 (48.2, 61.2)	**.04**	47.6 (42.7, 52.6)	55.8 (50.1, 61.5)	**<.01**
Sexually active scales (*N* = 157)^d^						
SA-CS	89.9 (88.2, 91.6)	86.5 (84.1, 88.9)	**<.01**	90.5 (88.8, 92.2)	86.6 (84.6, 88.7)	**<.001**
SA-CI	83.7 (80.6, 86.8)	77.6 (73.4, 81.7)	**<.01**	85.3 (82.2, 88.5)	76.6 (73.0, 80.2)	**<.001**
SA-GQ	64.6 (50.4, 68.9)	56.6 (51.6, 61.6)	**<.001**	65.2 (60.9, 69.6)	58.2 (53.6, 62.9)	**<.001**
**Fully-adjusted model** ^**e**^						
Non-sexually active scales (*N* = 125)^c^						
NSA-CS	15.1 (6.9, 23.2)	17.8 (8.6, 27.0)	.45	14.1 (5.8, 22.5)	18.7 (10.0, 27.4)	.16
NSA-CI	7.9 (-0.4, 16.2)	18.5 (9.2, 27.8)	**<.01**	11.4 (3.0, 19.9)	15.0 (6.2, 23.8)	.29
NSA-GQ	35.0 (24.6, 45.4)	44.8 (33.6, 56.1)	**.01**	38.0 (27.4, 48.5)	41.9 (31.1, 52.7)	.23
Sexually active scales (*N* = 151)^d^						
SA-CS	89.8 (85.0, 94.5)	86.2 (80.9, 91.6)	**.05**	89.5 (84.6, 94.5)	86.5 (81.4, 91.5)	.05
SA-CI	80.0 (70.4, 89.6)	79.0 (68.7, 89.4)	.72	82.0 (72.2, 91.8)	77.1 (67.1, 87.1)	**.04**
SA-GQ	67.3 (54.9, 79.7)	61.9 (48.8, 74.9)	.06	66.8 (54.2, 79.3)	62.4 (49.6, 75.2)	.09

a Mixed linear regression model taking into account within person correlations with repeated measures across different time points, adjusted for baseline age and intervention assignment.

b Minimally-adjusted models were adjusted for age and intervention assignment.

c For non-sexually active scales, higher scores indicate more interference or problems with sexual function. Because participants may have varied in their sexual activity status across visits, the *N* refers to the number of participants providing data on at least one timepoint.

d For sexually active scales, higher scores indicate better sexual function. Because participants may have varied in their sexual activity status across visits, the *N* refers to the number of participants providing data on at least one timepoint.

e Fully-adjusted models were adjusted for age, intervention assignment, self-reported race, BMI, menopausal status, and all other potentially influential factors (urinary incontinence type, urinary incontinence frequency, PROMIS Adult Physical Function Profile score, and 2-minute step test).

Abbreviations: HADS, Hospital Anxiety and Depression Scale; PISQ-IR, Prolapse/Incontinence Sexual Questionnaire, IUGA-Revised; PROMIS, Patient-Reported Outcomes Measurement Information System; NSA-CS, condition-specific reasons for not being active; NSA-CI, condition impact on sexual quality; NSA-GQ, global quality rating of sexual quality; SA-CI, condition-specific impact on sexual quality; SA-CS, assessment of condition-specific impacts on activity; SA-GQ, global quality rating of sexual quality.

## Discussion

Contrary to expectations, in this cohort of midlife and older women with at least daily UI, clinical incontinence characteristics did not emerge as major determinants of women’s sexual function. Neither incontinence type nor frequency was consistently associated with poorer outcomes after adjustment for other participant characteristics. Similarly, underlying physical function and performance were largely non-contributory to sexual function. Instead, the dominant factor associated with women’s sexual function was their psychological function. Greater depressive symptom burden and cognitive anxiety were consistently associated with worse sexual function across multiple domains in both sexually active and non-sexually active women with UI. While the direction of these relationships cannot be confirmed through this study design, these findings suggest that reductions in UI frequency may only contribute to improvements in sexual function if they are associated with concurrent improvements in psychological health.

Earlier analysis of data from this clinical trial demonstrated improvements in UI frequency among women in both intervention groups^12^; however, only modest improvements in sexual function were detected.^27^ In addition, a recent multicenter observational study of women seeking care for lower urinary tract symptoms (LUTS), including UI, demonstrated that although LUTS improved over time, sexual function did not.^28^ This current study provides greater insight into these findings. Because sexual function is multidimensional even among women with UI, improvement in sexual function may require multimodal treatment approaches that include attention to psychologic factors.

Prior studies of sexual function in women with UI have tended not to distinguish the relative importance of UI-related, physical, or psychological function-related contributors to sexual function in women with UI. Studies examining independent associations between UI severity and sexual function in women have yielded mixed results. For example, among pre-menopausal women seeking care at gynecology or urology practices, greater severity of UI was weakly but significantly associated with worse sexual function.^29^ However, a study of middle-aged women with stress UI participating in a trial of exercise interventions found no relation between the severity of UI and overall sexual function.^30^

In contrast, the association between UI, sexual function, and depression is well-documented,^6-10^^,^^31^^,^^32^ but the directionality of this relationship is unclear and is likely bidirectional. UI may lead women to experience low self-esteem and negative feelings toward their body image, impacting their psychologic health and sexual function, but there is also data suggesting that the underlying neurologic changes seen in depression may also influence voiding control leading to UI.^33^ Taken alongside past research, our results suggest that depression may help explain the link between UI and sexual function. Lastly, although there is evidence that declining physical function negatively impacts sexual function in women,^9^ we did not observe important relationships between either self-reported or objectively measured physical function and sexual function in this study sample. However, participants in our cohort were enrolled in a trial involving physical activities, and individuals with major mobility limitations were excluded.

Strengths of our study include its large and ethnically diverse sample, as well as its prospective design allowing for repeated assessments of sexual function, UI symptoms, physical function, and psychological function over time. This study also benefitted from the use of detailed, validated measures of sexual function, depression, anxiety, and physical function. However, limitations include the need to stratify analyses by sexual activity status to calculate sexual function scores. Such stratification may have reduced statistical power, potentially obscuring associations. Second, we examined associations between multiple types of participant characteristics and multiple domains of sexual function without formal adjustment for multiple comparisons, increasing the likelihood that some associations might appear significant by chance. Third, we did not have data about partner status or partner health and sexual functioning, which can influence one’s sexual activity and sexual function. Lastly, the study period overlapped with the COVID-19 pandemic, which may have limited participant’s opportunities for sexual activity.

## Conclusion

This study highlights the multiplicity of factors with the potential to influence, and be influenced by, sexual function in women with UI. Even for women with frequent or bothersome UI, addressing psychological health alongside UI symptoms may be essential to improve their sexual function. Given the complex relationship between psychological health, UI, and sexual function, these findings may empower clinicians caring for women with UI and sexual function concerns to recognize and treat these concerns as interconnected but distinct processes. They also support the need to develop and test more multimodal treatment approaches for women with UI who desire improved sexual function.

## IRB approval number

18-26 341.

## Supplementary Material

Supplementary_material_qdag175

## Notes

**Meeting Presentation**: This work was presented at the 2023 CAIRIBU (Collaborating for the Advancement of Interdisciplinary Research in Benign Urology) Annual Meeting.

## References

[1] Minassian VA, Stewart WF, Wood GC. Urinary incontinence in women: variation in prevalence estimates and risk factors. Obstet Gynecol. 2008;111(2):324–331. 10.1097/01.AOG.0000267220.48987.17 18238969

[2] Duralde ER, Rowen TS. Urinary incontinence and associated female sexual dysfunction. Sex Med Rev. 2017;5(4):470–485. 10.1016/j.sxmr.2017.07.001 28827036

[3] Schoenfeld M, Fuermetz A, Muenster M, et al. Sexuality in German urogynecological patients and healthy controls: is there a difference with respect to the diagnosis? Eur J Obstet Gynecol Reprod Biol. 2013;170(2):567–570. 10.1016/j.ejogrb.2013.08.002 23988220

[4] Felippe MR, Zambon JP, Girotti ME, et al. What is the real impact of urinary incontinence on female sexual dysfunction? A Case Control Study. Sex Med. 2017;5(1):e54–e60. 10.1016/j.esxm.2016.09.001 28087237 PMC5302384

[5] Pinheiro Sobreira Bezerra LR, Britto DF, Ribeiro Frota IP, et al. The impact of urinary incontinence on sexual function: a systematic review. Sex Med Rev. 2020;8(3):393–402. 10.1016/j.sxmr.2019.06.009 32409182

[6] Atlantis E, Sullivan T. Bidirectional association between depression and sexual dysfunction: a systematic review and meta-analysis. J Sex Med. 2012;9(6):1497–1507. 10.1111/j.1743-6109.2012.02709.x 22462756

[7] Milsom I, Kaplan SA, Coyne KS, Sexton CC, Kopp ZS. Effect of bothersome overactive bladder symptoms on health-related quality of life, anxiety, depression, and treatment seeking in the United States: results from EpiLUTS. Urology. 2012;80(1):90–96. 10.1016/j.urology.2012.04.004 22748867

[8] Saleh S, Almadani N, Mahfouz R, Nofal H, El-Rafey D, Seleem D. Exploring the intersection of depression, anxiety, and sexual health in perimenopausal women. Int J Womens Health. 2024;16:1315–1327. 10.2147/IJWH.S464129 39100112 PMC11298183

[9] Appa AA, Creasman J, Brown JS, et al. The impact of multimorbidity on sexual function in middle-aged and older women: beyond the single disease perspective. J Sex Med. 2014;11(11):2744–2755. 10.1111/jsm.12665 25146458 PMC4309673

[10] Tavoli A, Tavoli Z, Effatpanah M, Montazeri A. Prevalence and associated risk factors for sexual dysfunction among postmenopausal women: a study from Iran. Womens Midlife Health. 2021;7(1):10. 10.1186/s40695-021-00069-0 34838138 PMC8626990

[11] Matthias RE, Lubben JE, Atchison KA, Schweitzer SO. Sexual activity and satisfaction among very old adults: results from a community-dwelling medicare population survey. Gerontologist. 1997;37(1):6–14. 10.1093/geront/37.1.6 9046699

[12] Huang AJ, Chesney M, Schembri M, et al. Efficacy of a therapeutic pelvic yoga program versus a physical conditioning program on urinary incontinence in women: a randomized trial. Ann Intern Med. 2024;27:M23–M3051. Published online August. 10.7326/M23-3051 PMC11473233 39186785

[13] Rogers RG, Rockwood TH, Constantine ML, et al. A new measure of sexual function in women with pelvic floor disorders (PFD): the pelvic organ prolapse/incontinence sexual questionnaire, IUGA-revised (PISQ-IR). Int Urogynecol J. 2013;24(7):1091–1103. 10.1007/s00192-012-2020-8 23632798

[14] Grzybowska ME, Wydra D. Responsiveness of two sexual function questionnaires: PISQ-IR and FSFI in women with pelvic floor disorders. Neurourol Urodyn. 2021;40(1):358–366. 10.1002/nau.24568 33150611

[15] Locher JL, Goode PS, Roth DL, Worrell RL, Burgio KL. Reliability assessment of the bladder diary for urinary incontinence in older women. J Gerontol Ser A Biol Med Sci. 2001;56(1):M32–M35. 10.1093/gerona/56.1.M32 11193230

[16] Brown JS, McNaughton KS, Wyman JF, et al. Measurement characteristics of a voiding diary for use by men and women with overactive bladder. Urology. 2003;61(4):802–809. 10.1016/S0090-4295(02)02505-0 12670569

[17] Rose M, Bjorner JB, Becker J, Fries JF, Ware JE. Evaluation of a preliminary physical function item bank supported the expected advantages of the patient-reported outcomes measurement information system (PROMIS). J Clin Epidemiol. 2008;61(1):17–33. 10.1016/j.jclinepi.2006.06.025 18083459

[18] Rikli RE, Jones CJ. Development and validation of criterion-referenced clinically relevant fitness standards for maintaining physical independence in later years. Gerontologist. 2013;53(2):255–267. 10.1093/geront/gns071 22613940

[19] Radloff LS . The CES-D scale: a self-report depression scale for research in the general population. Appl Psychol Meas. 1977;1(3):385–401. 10.1177/014662167700100306

[20] Zigmond AS, Snaith RP. The hospital anxiety and depression scale. Acta Psychiatr Scand. 1983;67(6):361–370. 10.1111/j.1600-0447.1983.tb09716.x 6880820

[21] Rogers R, Bachmann G, Jumadilova Z, et al. Efficacy of tolterodine on overactive bladder symptoms and sexual and emotional quality of life in sexually active women. Int Urogynecol J. 2008;19(11):1551–1557. 10.1007/s00192-008-0688-6 18685795

[22] Yoshida M, Takeda M, Gotoh M, et al. Efficacy of vibegron, a novel β3-adrenoreceptor agonist, on severe urgency urinary incontinence related to overactive bladder: post hoc analysis of a randomized, placebo-controlled, double-blind, comparative phase 3 study. BJU Int. 2020;125(5):709–717. 10.1111/bju.15020 31991511 PMC7318146

[23] Huang AJ, Walter LC, Yaffe K, et al. TReating Incontinence for Underlying Mental and Physical Health (TRIUMPH): a study protocol for a multicenter, double-blinded, randomized, 3-arm trial to evaluate the multisystem effects of pharmacologic treatment strategies for urgency-predominant urinary incontinence in ambulatory older women. Trials. 2023;24(1):287. 10.1186/s13063-023-07279-z 37085880 PMC10122333

[24] Fomenko A, Dümmler D, Aktürk Z, et al. Hospital Anxiety and Depression Scale Anxiety subscale (HADS-A) for detecting anxiety disorders in adults. Cochrane Central Editorial Service, ed. Cochrane Database of Syst Rev. 2025;2025(7):1–215. 10.1002/14651858.CD015456 PMC12216811 40600405

[25] Bachman SL, Gomes E, Aryal S, et al. Do measures of real-world physical behavior provide insights into the well-being and physical function of cancer survivors? Cross-sectional analysis. JMIR Cancer. 2024;10:e53180. 10.2196/53180 39008350 PMC11287100

[26] Oursler KK, Briggs BC, Lozano AJ, Harris NM, Marconi VC, Ryan AS. Association of step count with cardiorespiratory fitness: results from the virtual 2-minute step test. Arch Rehabil Res Clin Transl. 2024;6(4):100369. 10.1016/j.arrct.2024.100369 39822200 PMC11734009

[27] Yang N, Subak LL, Shatkin-Margolis A, et al. Sexual function in a randomized trial of pelvic yoga for urinary incontinence. Urogynecol. 2025;32:635–645. 10.1097/SPV.0000000000001705 PMC12673998 41329612

[28] Bretschneider CE, Smerdon C, Bieber B, et al. Sexual function in women with bothersome lower urinary tract symptoms: findings from the symptoms of lower urinary tract dysfunction research network (LURN) cohort study. J Sex Med. 2025;22(6):1035–1042. 10.1093/jsxmed/qdaf063 40269441

[29] Serenko A, Morrison B, Suresky J. Urinary incontinence and sexual function in pre-menopausal women. Int J of Uro Nursing. 2010;4(2):80–86. 10.1111/j.1749-771X.2010.01098.x

[30] Liebergall-Wischnitzer M, Paltiel O, Hochner-Celnikier D, Lavy Y, Manor O, Woloski Wruble AC. Sexual function and quality of life for women with mild-to-moderate stress urinary incontinence. J Midwife Womens Health. 2011;56(5):461–467. 10.1111/j.1542-2011.2011.00076.x 23181643

[31] Abebe SA, Gashaw F, Tsegaye A, Abebaw D, Kindie EA, Dejen AM. Prevalence and determinants of depression among women with urinary incontinence: a systematic review and meta-analysis worldwide. BMC Womens Health. 2024;24(1):591. 10.1186/s12905-024-03432-1 39501253 PMC11539296

[32] Lai HH, Shen B, Rawal A, Vetter J. The relationship between depression and overactive bladder/urinary incontinence symptoms in the clinical OAB population. BMC Urol. 2016;16(1):60. 10.1186/s12894-016-0179-x 27716241 PMC5053341

[33] Littlejohn JO, Kaplan SA. An unexpected association between urinary incontinence, depression and sexual dysfunction. Drugs Today (Barc). 2002;38(11):777–782. 10.1358/dot.2002.38.11.740199 12582461

