# Supplementary material for: Beyond the bladder: incontinence-specific, physical, and psychological contributors to sexual function in women with urinary incontinence
Source: J Sex Med. 2026 Jun 25;23(7):qdag175. doi: 10.1093/jsxmed/qdag175 (PMC13298851; doi:10.1093/jsxmed/qdag175)
Supplement: Supplementary_material_qdag175 [file supplementary_material_qdag175.zip › supplementalmateral_20260327.docx]

Supplemental Table 1: Sexual activity status by timepoint

|  | **Baseline** | **6 weeks** | **12 weeks** | **24 weeks** | **36 weeks** |
| --- | --- | --- | --- | --- | --- |
| Not sexually active | 107 (45.3) | 89 (41.6) | 84 (40.4) | 62 (37.3) | 52 (36.6) |
| Sexually active | 129 (54.7) | 125 (58.4) | 124 (59.6) | 104 (62.7) | 90 (63.4) |
| Total | 236 | 214 | 208 | 166 | 142 |

Supplemental Table 2: Mean baseline Pelvic Organ Prolapse/Incontinence Sexual Questionnaire, IUGA-Revised (PISQ-IR) domain scores

| **Pelvic Organ Prolapse/Incontinence Sexual Questionnaire, IUGA-Revised domain** | **Mean (±SD)** |
| --- | --- |
| Non-sexually active |  |
| Condition-specific reasons for not being active (NSA-CS) | 22.4 (±22.1) |
| Condition impact on sexual quality (NSA-CI) | 17.8 (±24.7) |
| Global quality (NSA-GQ) | 49.1 (±30.9) |
| Sexually active |  |
| Assessment of condition-specific impacts on activity (SA-CS) | 86.6 (±13.3) |
| Condition impact on sexual quality (SA-CI) | 80.4 (±21.8) |
| Global quality (SA-GQ) | 63.2 (±28.1) |
